# Prospective Uncontrolled Interventional Study of Itraconazole and β-Glucans (*Euglena gracilis*) to Assess Safeness and Clinical Effectiveness in Cats with Cutaneous and Mucosal Sporotrichosis

**DOI:** 10.3390/vetsci12090830

**Published:** 2025-08-28

**Authors:** André Felipe Pedrazzi Chacon, Anna Barreto Fernandes Figueiredo, Jéssica Sepulveda Boechat, Erica Guerino Reis, Cindy Caroline dos Santos Honorato, Maria Lopes Corrêa, Sandro Antonio Pereira, Isabella Dib Ferreira Gremião

**Affiliations:** 1 Laboratório de Pesquisa Clínica em Dermatozoonoses em Animais Domésticos (Lapclin-Dermzoo), Instituto Nacional de Infectologia Evandro Chagas (INI), Fundação Oswaldo Cruz (Fiocruz), Rio de Janeiro 21040-360, Brazil; andre.chacon@ini.fiocruz.br (A.F.P.C.); anna.figueiredo@ini.fiocruz.br (A.B.F.F.); jessica.boechat@ini.fiocruz.br (J.S.B.); cindy.honorato@ini.fiocruz.br (C.C.d.S.H.); maria.correa@ini.fiocruz.br (M.L.C.); sandro.pereira@ini.fiocruz.br (S.A.P.); 2 Coordenação de Uma Só Saúde, Instituto de Ciência e Tecnologia em Biomodelos (ICTB), Fundação Oswaldo Cruz (Fiocruz), Rio de Janeiro 21040-360, Brazil; erica.reis@fiocruz.br

**Keywords:** *Sporothrix*, cats, therapy, itraconazole, β-glucans

## Abstract

Brazil is an endemic region for both human and feline sporotrichosis, with *Sporothrix brasiliensis* being the main etiological agent. Currently, few effective antifungal agents are available for treating this mycosis in cats, and therapeutic studies remain limited. Itraconazole (ITZ) is the first-line drug; however, its effectiveness is variable. To evaluate the use of ITZ combined with β-glucans (*Euglena gracilis*) in feline sporotrichosis, a prospective, uncontrolled interventional study was conducted in 29 cats. Clinical cure was achieved in 21 animals (72.4%) with a median treatment duration of 10 weeks. Most of these cats presented with nasal region lesions, nasal mucosa involvement, and respiratory signs, which are commonly associated with poor therapeutic outcomes. Treatment failure occurred in 5 animals (17.2%), and 3 (10.3%) were lost to follow-up. No deaths were recorded during the study. Adverse drug reactions (ADRs) were observed in 2 cats (6.9%). These findings suggest that β-glucans may be a complementary strategy in the treatment of feline sporotrichosis, particularly in cases involving nasal lesions and respiratory signs, and may also contribute to the prevention of ADRs associated with conventional therapy.

## Simple Summary

Sporotrichosis is a fungal disease that affects both humans and cats, with a high occurrence in Brazil. Treating cats can be difficult because there are few effective drugs available, and the commonly used one does not always work well. In this study, 29 cats with sporotrichosis were treated with the usual drug (itraconazole) combined with a natural substance called beta-glucans, which may help the immune system. Most cats (about 72%) were cured within 10 weeks. Some cats had lesions in their noses and respiratory problems, which often makes treatment harder. A small number of cats did not get better or were lost to follow-up during the study, and side effects were rare. These results suggest that adding beta-glucans to itraconazole might help cats recover better and reduce side effects. This could improve care for cats with sporotrichosis and help prevent the spread of the disease.

## 1. Introduction

Feline sporotrichosis is a subcutaneous mycosis caused by pathogenic species of the genus *Sporothrix*. The etiological agents identified in cats include *S. brasiliensis*, *S. schenckii*, *S. globosa*, *S. humicola* [1] and *S. davidellisii* [2]. *S. schenckii* is the main agent in most parts of the world, except in Brazil, where *S. brasiliensis* is the most prevalent species [1]. In this country, high rates of human and feline cases caused by *S. brasiliensis* have been reported since the late 1990s, with Rio de Janeiro considered the epicenter of this epidemic [1,3,4,5,6,7,8,9].

Feline sporotrichosis can be acquired through traumatic implantation from environmental sources such as plant material, soil, and decaying wood. Transmission can also occur through contact with infected cats, primarily via bites, scratches, or exposure to lesion exudates and respiratory droplets [1,10]. Cat-transmitted sporotrichosis poses significant public health concerns, particularly in South America and Malaysia [1,11,12,13,14], where cats act as important sources of infection, transmitting the fungus to other cats, humans, and, less frequently, dogs. In other regions, clusters of zoonotic cases associated with cats remain uncommon; however, recent reports from the United States, Australia, Thailand, and the United Kingdom indicate both the emergence and reemergence of *Sporothrix* spp. [15,16,17,18].

Sporotrichosis occurs more frequently in young adult, mixed-breed, unneutered male cats. Clinical presentations range from a single localized lesion to multiple lesions, with some cases progressing to fatal systemic dissemination. Nasal mucosal involvement is frequent and commonly associated with respiratory signs such as sneezing, inspiratory dyspnea, and nasal discharge. Lymphadenomegaly is a typical finding, whereas lymphangitis is less frequently documented [19,20]. The incubation period of the disease generally ranges from 3 to 30 days, although in some instances it may extend for several months [21].

To date, few effective antifungal drugs are available for the treatment of this mycosis in cats, and therapeutic studies remain scarce. Although itraconazole (ITZ) remains the first-line therapy for feline sporotrichosis, accumulating evidence indicates that this recommendation should be considered with nuance. Reported cure rates have varied substantially across studies, ranging from 77% in naïve cats [22] to moderate outcomes of approximately 50% in more recent investigations [20,23]. This pattern, combined with reports of prolonged or unsuccessful ITZ monotherapy [24,25] and the frequent need for combination with KI in refractory cases [26], highlights the need to reassess its role as a universal first-line option. In particular, the presence of negative prognostic indicators may justify further evaluation of upfront combination strategies [20]. In Brazil, the combination of ITZ and potassium iodide (KI) has become an important option for feline cases unresponsive to ITZ monotherapy [20,26].

The main adverse reactions associated with the treatment using ITZ, whether as monotherapy or in combination with KI, are well documented in the literature. These include gastrointestinal disturbances, such as diarrhea, anorexia, vomiting, and hyporexia, as well as elevations in liver enzymes [20,27].

Other systemic antifungal agents described for the treatment of feline sporotrichosis include ketoconazole, KI as monotherapy, sodium iodide, fluconazole, terbinafine, posaconazole, isavuconazole, and amphotericin B. Adjunctive therapies include local heat therapy, laser therapy, photodynamic therapy, cryosurgery, surgical excision of lesions, and intranasal clotrimazole spray [19,21,25,27,28,29,30,31,32,33,34,35,36,37,38].

Multiple factors can influence the therapeutic response in feline sporotrichosis. Treatment success depends not only on the host-pathogen interaction and owner adherence, but also on therapy-related variables such as drug formulation, administration practices, and the pharmacokinetics of antifungal agents [21,39]. Resistance to ITZ in *Sporothrix* spp. has become a concern due to increasing reports of therapeutic failure and the emergence of isolates with high minimum inhibitory concentrations (MICs) [1,40]. However, a consistent correlation between MIC values and clinical response has not been established, and antifungal susceptibility results should therefore be interpreted within the broader clinical context [41].

Beyond the variability in reported cure rates, studies have provided insights into why ITZ monotherapy often fails. Histopathological analyses of refractory nasal lesions have revealed persistent fungal structures and scarce immune cell infiltration despite prolonged therapy [42], suggesting that factors beyond systemic drug exposure, such as localized immune dysregulation may contribute to treatment failure. In a recent study, higher neutrophil oxidative burst was associated with good clinical condition and favorable treatment outcomes, whereas cats with nasal mucosal involvement exhibited reduced neutrophil activation, a finding linked to therapeutic failure [23]. Earlier immunophenotyping studies also showed that severe forms, with high fungal burden and widespread lesions, were linked to an increase of CD8^low^ cells and depletion of CD4+ cells [43], indicating combined defects in innate and adaptive responses. Collectively, these findings suggest that ITZ failure is multifactorial, involving fungal tolerance, tissue penetration barriers, and impaired host immunity. This complexity strengthens the rationale for host-directed strategies such as β-glucans, which may enhance immune responsiveness while also offering hepatoprotective benefits.

In light of these challenges, there has been growing interest in exploring adjunctive strategies capable of enhancing the host immune response to improve treatment outcomes. β-glucans are naturally occurring polysaccharides found in the cell walls of yeasts, mushrooms, algae, bacteria, barley, and oats [44]. These compounds have well-documented immunomodulatory properties, stimulating key components of both innate and adaptive immunity. They have been investigated in diverse contexts, including solid tumors, hematological malignancies, immune-mediated disorders and wound healing [45,46,47].

The unicellular alga *Euglena gracilis* is a notable source of β-1,3-glucan (paramylon), which forms crystalline granules under specific growth conditions [48]. Paramylon has shown immunomodulatory effects, including reducing atopic dermatitis lesions in mice [49], and fermented *E. gracilis* supplementation (>50% β-1,3-glucan) has been associated with reduced respiratory infection symptoms and improved immune health in humans [50].

In veterinary medicine, studies investigating *E. gracilis* have been reported in dogs, including its use as an adjuvant in chronic kidney disease [51] and its ability to modulate immune and inflammatory responses [52]. In cats, β-glucans from other sources have been tested in clinical settings, such as chronic kidney disease, dermatologic conditions, osteoarthritis, and inflammatory bowel disease [53,54]. Despite this evidence, no studies have evaluated β-glucans derived specifically from *E. gracilis* in cats, nor investigated their application in feline sporotrichosis. Therefore, this work represents the first exploration of this promising therapeutic approach.

In this context, there is a clear need for alternative or adjunctive therapeutic strategies that can overcome the limitations of ITZ monotherapy and reduce the adverse reactions associated with the ITZ + KI regimen. Despite their documented immunomodulatory and hepatoprotective properties in other species and clinical conditions, β-glucans from *E. gracilis* have never been investigated in feline sporotrichosis. This represents a critical knowledge gap, particularly given the high rates of treatment failure and the zoonotic implications of non-resolving infections. Therefore, the present study aims to evaluate the safeness and clinical effectiveness of the combined treatment of ITZ with β-glucans from *E. gracilis* under clinical practice conditions providing novel insights that may expand treatment options and improve clinical outcomes for feline sporotrichosis, with potential benefits for both veterinary practice and public health.

## 2. Materials and Methods

This was a prospective, uncontrolled interventional study, conducted between 2023 and 2024 at Laboratório de Pesquisa Clínica em Dermatozoonoses em Animais Domésticos (Lapclin-Dermzoo), Instituto Nacional de Infectologia Evandro Chagas (INI), Fundação Oswaldo Cruz (Fiocruz). The study protocol and informed consent form were approved by the Institutional Ethics Commission on the Use of Animals (CEUA/Fiocruz), under license number LW26/23.

### 2.1. Inclusion, Exclusion and Elimination Criteria

Cats aged between 6 months and 8 years, weighing at least 3 kg, and showing clinical signs suggestive of sporotrichosis were included. Exclusion criteria comprised prior treatment with oral antifungals or corticosteroids, as well as pregnancy or lactation. Cats with negative fungal culture results or whose owners declined treatment were withdrawn from the study.

### 2.2. Study Procedures and Techniques

All cats enrolled in the study were evaluated monthly through clinical examination, collection of clinical specimens, and photographic documentation. A detailed anamnesis was performed at each follow-up visit. Information provided by the owners was used to estimate the duration between the appearance of clinical signs and the initial clinical evaluation.

At the first visit, samples for fungal culture were collected from ulcerated cutaneous lesions using a sterile swab and seeded onto Sabouraud dextrose agar and Mycosel agar (Difco™; Becton, Dickinson and Company, Sparks Glencoe, MD, USA), incubated at 25 °C, and observed for four weeks for fungal growth. Dimorphism was demonstrated by conversion to the yeast-like form on brain heart infusion agar medium (Difco™) at 37 °C [1].

The presence of cutaneous and/or mucosal lesions, extracutaneous signs (lymphadenomegaly and respiratory signs), and the animal’s overall condition (good, fair, or poor) were evaluated during clinical examination. To estimate the level of dissemination of cutaneous lesions, the cats were divided into three groups: L1 (cutaneous lesions at one site), L2 (cutaneous lesions at two non-adjacent sites), and L3 (cutaneous lesions at three or more non-adjacent sites) [19].

Blood samples were collected for serum biochemistry evaluation (urea, creatinine, alanine aminotransferase [ALT], aspartate aminotransferase [AST], alkaline phosphatase [ALP], and gamma-glutamyl transferase [GGT]).

### 2.3. Treatment

The research team supervised the treatment process throughout the study, with evaluations conducted at each follow-up visit.

The cats were treated with ITZ capsule (100 mg per cat; Traxonol^®^, Geolab, Anápolis, GO, Brazil) and β-glucan derived from *E. gracilis* (Refos Derme^®^, Avert, Bragança Paulista, SP, Brazil) at a dose of one tablet per 10 kg, both administered orally once daily (every 24 h) until the end of treatment. The dosing regimen of β-glucan followed the manufacturer’s recommendations.

Owners were instructed to administer both drugs simultaneously, either directly into the oral cavity or mixed with small amount of canned cat food. They were also advised to contact the veterinary staff if signs of hepatotoxicity occurred, such as hyporexia, vomiting, or diarrhea. All procedures and drugs were provided free of charge.

For the purposes of this study, the cats were followed up for a maximum period of 5 months predefined based on the median treatment duration observed in previous studies using ITZ monotherapy [20]. Treatment outcomes were classified as clinical cure, treatment failure, treatment abandonment, or death. Clinical cure was defined as complete resolution of cutaneous and/or mucosal lesions and remission of clinical signs. In such cases, treatment was continued for an additional month. Therapeutic failure was defined as the lack of clinical improvement at a subsequent follow-up visit; these cats were then monitored as part of routine clinical care at Lapclin-Dermzoo/INI/Fiocruz. Treatment abandonment was defined as failure to attend a consecutive follow-up visit.

### 2.4. Monitoring of Adverse Drug Reactions (ADRs)

Adverse clinical reactions and laboratory abnormalities were evaluated in the follow-up appointments. Cats presenting anorexia or hyporexia combined with body loss of >10% or the association of adverse clinical reactions and elevated serum aminotransferase had their treatment interrupted temporarily for a minimum of 7 days and received hepatoprotective therapy consisting of oral silymarin (30 mg/kg/day; compounded formulation) until the end of the study [20].

Laboratory abnormalities were classified as mild (serum transaminase levels less than two times the upper limit of the reference range), moderate (serum transaminase levels two to five times the upper limit of the reference range), or marked (serum transaminase levels greater than five times the upper limit of the reference range) [55].

### 2.5. Statistical Analysis

An exploratory analysis of the database was performed, along with univariate and bivariate analyses. For continuous variables (age and weight), and measures of central tendency (median) were used.

Frequency analysis was performed for categorical variables, including sex, neuter status, overall condition, distribution of cutaneous lesions, cutaneous lesions on the nasal region, mucosal lesions, respiratory signs, ADRs, time until clinical cure, and outcomes.

A time-to-event analysis was performed using Kaplan–Meier survival curves to evaluate clinical cure. Survival distributions were compared with the log-rank test across three variables: presence or absence of mucosal lesions, neuter status, and outdoor access. Statistical significance was defined as *p* < 0.05.

All analyses were performed using R statistical software, version 4.4.2 (R Foundation for Statistical Computing, Vienna, Austria).

## 3. Results

A total of 92 cats were evaluated for eligibility. Of these, 42 cats with suspected sporotrichosis met the inclusion criteria and were initially enrolled. Among them, 13 (31.0%) were withdrawn from the study, either due to a negative fungal culture (5; 11.9%) or owner refusal (8; 19.1%). The remaining 29 (69.0%) were followed until a final outcome was reached.

All animals were mixed-breed cats from metropolitan region of Rio de Janeiro. Most were male (25; 86.2%) and in good overall condition (21; 72.4%). The median age was 33 months (range: 11–72 months), and the median weight was 4.3 kg (range: 3.0–5.7 kg). More than half of the cats (17; 58.2%) had outdoor access. The time interval between the onset of clinical signs ranged from 1 to 48 weeks, with a median of 4 weeks.

Ulcerative cutaneous lesions were observed in all animals. The cephalic region was the most affected anatomical site (24; 82.7%), and 15 (51.7%) had lesions on the nasal area. Mucosal involvement was noted in 19 (65.5%), predominantly affecting the nasal mucosa (18; 62.1%), of which 4 (4; 13,8%) also presented conjunctival lesions. Additionally, 1 cat exhibited exclusive conjunctival involvement (1; 3,4%). Respiratory signs, including sneezing, rhinorrhea, or inspiratory dyspnea, were reported in 17 (58.6%). Only one cat exhibited nodular ascending lymphangitis.

### Treatment Outcome

**Figure 1:**
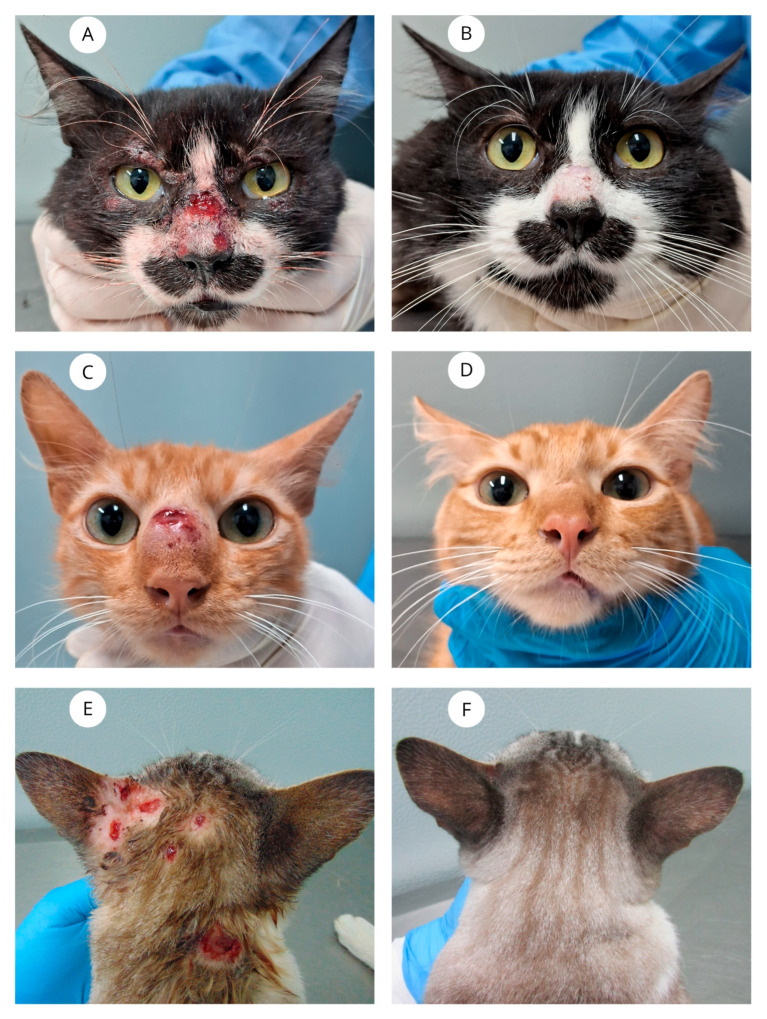
(**A**) Cat with sporotrichosis presenting ulcers on the nasal planum and nasal bridge, and lesions in the periorbital regions. (**B**) All lesions have resolved at 16 weeks of ITZ + β-glucan therapy. (**C**) Cat with sporotrichosis with an ulcerated nodule on the nasal bridge. (**D**) The lesion has resolved at 13 weeks of treatment. (**E**) Cat with sporotrichosis presenting multiple ulcers on the cephalic and cervical regions. (**F**) All lesions have resolved at 12 weeks of treatment.

Clinical cure was achieved in 21 cats (72.4%), with a median time to cure of 10 weeks (range: 4–19 weeks) (Figure 1). After achieving clinical cure, therapy was maintained for approximately one additional month.

**Figure 2:**
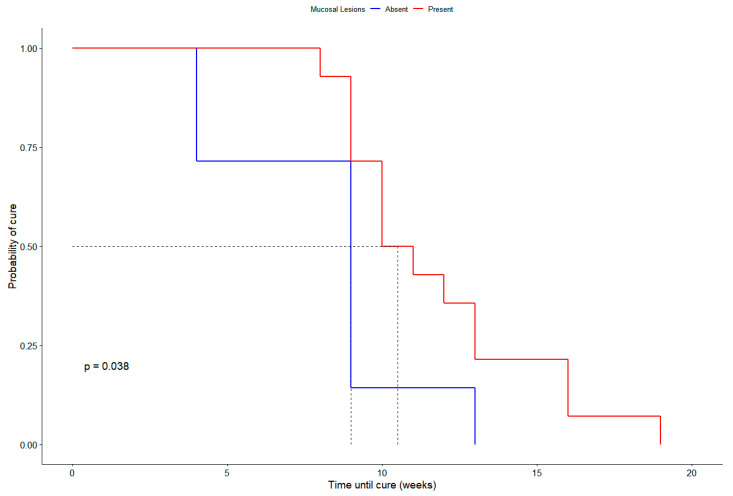
Kaplan–Meier survival curves showing the time to clinical cure in cats with sporotrichosis, stratified by mucosal involvement. The dashed horizontal line represents the 50% probability of cure, and the dashed vertical lines indicate the median time to cure for each group. Cats with mucosal lesions (red line) had a significantly longer time to cure compared to those without mucosal involvement (blue line) (log-rank test, *p* = 0.038).

Kaplan–Meier survival analyses were performed to explore prognostic variables associated with time to clinical cure. Cats with mucosal involvement had a significantly longer time to cure compared to those without (log-rank test, *p* = 0.038) (Figure 2). Neutered cats reached clinical cure significantly earlier than non-neutered cats (log-rank test, *p* = 0.016). Outdoor access was not associated with differences in time to cure (log-rank test, *p* = 0.86).

Failure occurred in 5 (17.2%), of which 3 (10.3%) presented with respiratory signs, nasal mucosal lesions, cutaneous lesions at three or more sites (L3), and fair overall condition. Three cats (10.3%) were lost to follow-up: two escaped, and one had scheduled visits discontinued by the owner despite repeated contact attempts. No deaths occurred during the study period.

Regarding the administration of ITZ by the owners, 17 (58.6%) received this drug as an opened capsule mixed with food, while 12 (41.4%) received a closed capsule.

ADRs were observed in 2 cats (6.9%) within the first month of treatment. One cat experienced anorexia, vomiting, and weight loss, while the other presented anorexia and weight loss associated with mild ALT and AST elevation. In both cases, treatment was temporarily suspended for 7 days, and oral silymarin was maintained throughout the study period. The clinical, laboratory and therapeutic characteristics of all cats are described in Table 1.

## 4. Discussion

The role of β-glucans in fungal infections remains poorly explored. Given the challenges in managing feline sporotrichosis, this study evaluated the potential benefits of combining β-glucans with ITZ in this context.

In cats with sporotrichosis treated with ITZ, clinical cure rates vary widely, ranging from 38.8% to 100% [20,22,29,56]. In the present study, a high clinical cure rate was observed (72.5%), especially considering that most animals presented with nasal region lesions, nasal mucosa involvement and respiratory signs, manifestations commonly associated with treatment failure [20,29]. Moreover, the median time to clinical cure was 10 weeks, compared to 14.9 weeks observed with ITZ monotherapy in a previous clinical trial conducted under similar clinical settings [20]. Although the cellular response was not evaluated in this study, β-glucans may have contributed to the healing process by modulating the immune response, including enhanced recruitment of macrophages and neutrophils and the activation of their functions, such as phagocytosis, oxidative burst, and cytokine production [46,47,57,58,59,60]. Further evidence of their therapeutic potential comes from studies in humans, where β-glucans act as adjuvants in preventing symptoms of allergic rhinitis and upper respiratory tract infections [50]. Although β-glucans may contribute to immune modulation, it is important to note that this study was not designed to investigate the pharmacological activities of β-glucan or ITZ. Instead, our focus was on clinical outcomes in naturally infected cats under clinical practice conditions. Future studies specifically addressing pharmacological mechanisms and immunomodulatory effects will be valuable to complement these findings.

The time between the appearance of cutaneous lesions and the initial clinical visit in this study was shorter (median: 4 weeks) than that reported in previous studies (median: 8 weeks) [19,20], a factor that could explain the more favorable prognosis observed. Early diagnostic investigation of suspected cases is critical to enable prompt initiation of antifungal treatment, reduce fungal burden, and potentially decrease transmission rates [22]. Moreover, prognosis can be significantly influenced by the cat’s overall condition and by the number, extent, and location of lesions [21], underscoring the importance of early treatment in feline sporotrichosis.

To reduce owner contact with sick animals and minimize the risk of zoonotic transmission, administering ITZ as an opened capsule mixed with food is recommended [21]. Pereira-Oliveira et al. [39] demonstrated that the mode of administration of ITZ monotherapy (opened vs. closed capsule) does not affect therapeutic plasma concentrations in cats, and that most treated cats achieved clinical cure. In our study, most cats received ITZ as opened capsules mixed with food and achieved favorable clinical outcomes.

There are few reports on the use of β-glucans as adjuncts in the treatment of cutaneous diseases in dogs and cats. A dog with refractory demodicosis and secondary infections was treated with ivermectin, antibiotics, and daily β-glucan (20 mg), showing clinical and microscopic improvement after two months; however, the β-glucan source was not specified, and the improvements could not be solely attributed to it due to concurrent therapies [61]. In a refractory case of canine sporotrichosis, subcutaneous β-1,3-glucan (0.5 mg/animal weekly for 4 weeks) was administered following partial improvement with antibiotics and ITZ, resulting in negative cultures. The β-glucan source was also not reported [62]. A topical mixture containing β-glucan and other compounds was applied to six dogs with atopic dermatitis for 30 days, leading to reduced pruritus and lesion severity. No concurrent treatments were used, except for one dog receiving a monoclonal antibody [63]. In a cat presenting with post-traumatic necrosis of the skin and subcutaneous tissues, β-glucan was applied topically as a gel (with chlorhexidine digluconate and pure bee honey) once daily, alongside oral administration of β-1,3/1,6-glucan, ascorbic acid, and zinc. Lesions healed within six weeks, and healing time was reduced by 30% [64]. In contrast to those reports, the present study was a case series that employed a well-characterized β-glucan, administered at a recommended dose.

In our study, most cats that experienced treatment failure presented with respiratory signs, nasal mucosal involvement, cutaneous lesions affecting three or more sites (L3), and a fair overall condition. These clinical features have been previously recognized as prognostic markers, thereby underscoring their significance in predicting therapeutic outcomes in feline sporotrichosis [20,22,29], reinforcing their value as prognostic indicators of poor therapeutic response. Moreover, outdoor access could plausibly influence treatment outcomes by increasing the likelihood of repeated environmental exposure or reinfection during therapy, potentially raising fungal burden and delaying lesion resolution. In the present study, more than half of the cats had outdoor access, but Kaplan–Meier survival analysis with log-rank testing showed no significant difference in time to clinical cure between cats with and without outdoor access (*p* = 0.86). Similarly, previous data [20] did not identify a statistically significant effect of outdoor access on cure rates. Taken together, these findings suggest that while outdoor access is an important epidemiological factor, its direct impact on treatment outcomes remains unproven and warrants further investigation in larger trials.

The treatment abandonment rate was comparatively low, especially when contrasted with earlier studies, reporting rates ranging from 34% to 38.5% [19,65]. Consistent with our findings, Reis et al. [20] also observed a reduced abandonment rate, which may reflect the impact of updated therapeutic protocols and improved clinical management on adherence. This outcome may be related to the shorter treatment duration observed, as well as the good acceptability of Refos Derme^®^ tablets in cats, both factors likely contributing to improved compliance.

In our study, the incidence of ADRs was low (6.9%), with only one case of laboratory abnormality (3.5%). This rate is lower than those reported in previous studies, which described clinical ADRs in up to 42.2% of cats and elevated serum transaminases in 60% of cases during ITZ monotherapy [20], as well as gastrointestinal ADRs in 30.9% of cats [29]. This lower frequency of ADRs may be related to the use of β-glucans, which have demonstrated hepatoprotective properties, including the reduction of hepatic inflammation and oxidative stress, promotion of hepatocellular regeneration, prevention of fibrosis, and limitation of cellular necrosis [60,66,67]. The hepatoprotective effects of β-glucans derived from *E. gracilis* have also been confirmed in a murine model of carbon tetrachloride-induced acute liver injury [68], further supporting the potential of these compounds to mitigate ITZ-associated hepatotoxicity.

In Brazil, an important strategy for treating feline sporotrichosis is the combination of ITZ with KI. A study evaluating this regimen reported a higher clinical cure rate (88%); however, clinical ADRs were more frequent (47%), as well as laboratory abnormalities (51.8%) [20]. The use of β-glucans in conjunction with ITZ and KI may represent a promising therapeutic approach, especially in cases at higher risk for ADRs.

Although 92 cats were initially evaluated, only 42 (45.6%) met the inclusion criteria and were enrolled. Most exclusions were due to prior antifungal treatment, reflecting the epidemiological context of the metropolitan region of Rio de Janeiro, where feline sporotrichosis has been endemic for over two decades and cat owners are familiar with ITZ as the primary treatment, often using it empirically before seeking specialized care. Despite the limited sample size, the findings provide clinical insights and underscore the potential of combining ITZ with β-glucans in feline sporotrichosis.

## 5. Conclusions

Feline sporotrichosis has limited therapeutic options and treatment failures are frequent. In the population of cats studied the combination of ITZ with β-glucans derived from *E. gracilis* did not increase serum aminotransferase normal limits and develop anorexia, vomiting, and weight loss in most cats (93.1%). Overall, it was considered safe. Thus, this combination may also help reduce ADRs associated with conventional therapy. This treatment combination was also considered to be a potentially beneficial approach, especially in difficult-to-treat cases presenting with nasal lesions and respiratory signs. Further longitudinal studies with larger cohorts, standardized protocols, and immunological assessments are needed to validate these preliminary findings and to clarify the role of β-glucans as adjunctive agents in the management of this disease.

## Figures and Tables

**Table 1:** Clinical, epidemiological and therapeutic characteristics of 29 cats with sporotrichosis treated with ITZ + β-glucan at Lapclin-Dermzoo/INI/Fiocruz between 2023 and 2024.

Cats	Sex	Neutered	Overall Condition	Skin Lesions Distribution	Skin Lesions on the Nasal Region	Mucosal Lesions	Respiratory Signs	Adverse Drug Reactions	Time to Clinical Cure (weeks)	Outcomes
1	M	No	Good	L1	No	Nasal	Yes	No	13	Cure
2	M	No	Good	L3	No	Nasal	Yes	No	9	Cure
3	M	No	Fair	L3	Yes	Nasal, Conjuctival	Yes	No	16	Cure
4	M	No	Good	L2	Yes	No lesion	No	No	N/A	Abandonment
5	M	No	Good	L2	Yes	Nasal	No	No	10	Cure
6	M	No	Fair	L3	Yes	Nasal	Yes	No	N/A	Failure
7	M	Yes	Good	L3	Yes	Nasal, Conjuctival	Yes	No	N/A	Abandonment
8	M	No	Good	L2	Yes	Nasal	Yes	No	N/A	Abandonment
9	M	Yes	Good	L3	No	Conjuctival	Yes	No	8	Cure
10	M	No	Poor	L3	Yes	Nasal	Yes	No	19	Cure
11	M	No	Fair	L3	Yes	Nasal	Yes	No	N/A	Failure
12	F	No	Good	L2	No	No lesion	No	No	4	Cure
13	M	Yes	Good	L3	Yes	Nasal, Conjuctival	Yes	No	9	Cure
14	M	Yes	Good	L3	No	No lesion	No	No	9	Cure
15	M	Yes	Fair	L1	No	Nasal	No	No	9	Cure
16	M	Yes	Good	L2	No	Nasal, Conjuctival	Yes	No	12	Cure
17	M	No	Good	L1	No	No lesion	No	No	9	Cure
18	M	No	Fair	L3	Yes	Nasal	Yes	No	N/A	Failure
19	M	No	Good	L1	No	No lesion	No	No	9	Cure
20	M	Yes	Good	L3	Yes	Nasal	Yes	Yes *	10	Cure
21	M	Yes	Good	L1	No	No lesion	Yes	No	N/A	Failure
22	M	No	Good	L1	No	No lesion	No	No	13	Cure
23	F	Yes	Good	L1	Yes	Nasal	Yes	No	10	Cure
24	M	Yes	Good	L2	No	No lesion	No	No	N/A	Failure
25	M	No	Fair	L1	Yes	Nasal	Yes	No	13	Cure
26	M	No	Fair	L3	Yes	Nasal	No	No	16	Cure
27	F	Yes	Good	L1	No	No lesion	No	Yes **	9	Cure
28	M	Yes	Good	L1	No	No lesion	No	No	4	Cure
29	F	Yes	Good	L1	Yes	Nasal	Yes	No	11	Cure

* Anorexia, weight loss and mild ALT and AST elevation. ** Anorexia, vomiting, and weight loss. Abbreviations: M = Male; F = Female; L1 (skin lesions at one site); L2 (skin lesions at two non-adjacent sites); L3 (skin lesions at three or more non-adjacent sites); N/A = Not applicable.

## Data Availability

The data underlying this article will be shared on reasonable request to the corresponding author.
